# Oral contraceptive use is associated with smaller hypothalamic and pituitary gland volumes in healthy women: A structural MRI study

**DOI:** 10.1371/journal.pone.0249482

**Published:** 2021-04-21

**Authors:** Ke Xun Chen, Sandie Worley, Henry Foster, David Edasery, Shima Roknsharifi, Chloe Ifrah, Michael L. Lipton

**Affiliations:** 1 Department of Radiology, Albert Einstein College of Medicine and Montefiore Health, Bronx, NY, United States of America; 2 Department of Neurology, New York University School of Medicine, New York, NY, United States of America; 3 Gruss Magnetic Resonance Research Center, Albert Einstein College of Medicine and Montefiore Health, Bronx, NY, United States of America; 4 Department of Psychiatry and Behavioral Sciences, Albert Einstein College of Medicine and Montefiore Health, Bronx, NY, United States of America; 5 Dominick P. Purpura Department of Neuroscience, Albert Einstein College of Medicine and Montefiore Health, Bronx, NY, United States of America; University at Buffalo, UNITED STATES

## Abstract

The effects of hormonal contraceptives on structural features of the hypothalamus and pituitary are incompletely understood. One prior study reported microstructural changes in the hypothalamus with oral contraceptive pill (OCP) use. However, effects on hypothalamic volume have not been reported. One prior study reported volumetric changes in the pituitary. However, this study was limited by including participants evaluated for neurological symptoms. We sought to determine if OCP use is associated with alteration of hypothalamic or pituitary volume. High-resolution 3T MRI was performed for a prospective cohort of 50 healthy women from 2016 to 2018, which comprised 21 OCP users (age, 19–29) and 29 naturally cycling women (age, 18–36). Participants were excluded if they were pregnant or had significant medical conditions including neurological, psychiatric, and endocrine disorders. After confirming reliability of the image analysis techniques, 5 raters independently performed manual segmentation of the hypothalamus and semi-automated intensity threshold-based segmentation of the pituitary using ITK-SNAP. Total intracranial volume was estimated using FreeSurfer. A general linear model tested the association of OCP use with hypothalamic and pituitary volumes. Hypothalamic (B = -81.2 ± 24.9, p = 0.002) and pituitary (B = -81.2 ± 38.7, p = 0.04) volumes in OCP users were smaller than in naturally cycling women. These findings may be related to interference with known trophic effects of sex hormones and suggest a structural correlate of central OCP effects.

## Introduction

Oral contraceptive pills (OCPs) have been in the US market for almost 60 years with at least 10 million users in the United States and 100 million users worldwide [1]. The pill is one of the most commonly used forms of birth control in the United States, in wide use among younger women [2]. In general, the use of OCPs declines with age as the prevalence of other forms of contraception such as sterilization increases. The use of OCPs at a young age might confer long-term consequences as some brain effects of OCPs may not be completely reversible [3, 4]. Although structural and functional brain changes have been associated with ovarian sex hormone variation during the normal menstrual cycle, very little is known about the effects of exogenous steroids, such as OCPs, on the brain. Only a handful of studies are available in literature [3]. Information on the potential effects of OCPs could be valuable to physicians as they discuss choice of contraception with patients and address their concerns. Moreover, research studies have inconsistently accounted for the potential effects of hormonal contraceptives. These includes studies addressing sex differences in adult brain structure, which may in part explain their mixed results [5, 6]. In medicine, studies that have reported changes in hypothalamic volume in schizophrenic patients have not accounted for OCP use [7–9]. Thus, the potential impact of OCPs on brain structure is germane to both clinical practice and research.

The brain is a dynamic organ and demonstrates structural plasticity, reorganizing its physical structure and modulating function in response to variations in endogenous ovarian sex hormones. Most studies reporting OCP effects on brain structure have employed voxel-based morphometry. OCP use during the active pill phase was associated with larger volumes of multiple brain regions, including the fusiform gyrus bilaterally, when compared to naturally cycling women (NCW) in the follicular or luteal phase [10]. However, a more recent study suggested that OCP use during the active pill phase might be associated with a smaller left fusiform gyrus, incongruent with the previous study [11]. Using a surface-based approach, one study showed that OCP use was associated with decreased thickness of the lateral orbitofrontal and posterior cingulate cortices [12]. Adding to the complexity of OCP effects on the brain, longer duration of OCP use may be associated with larger regional brain volumes and changes in brain volumes may be related to the androgenicity of the progestin component of a given OCP preparation [4, 13]. This in turn may produce downstream functional consequences [4]. Although researchers are becoming increasingly aware of these factors [14], there is presently a general lack of consensus regarding the brain effects of OCPs, which may be due to small sample sizes, differences in morphometry techniques, and heterogeneity in timing of imaging across the few studies that have addressed effects of OCPs on brain structure.

The hypothalamus is the source of signals that regulate the female reproductive cycle and is affected by endogenous as well as exogenous sex steroids. In addition to its central role in sex hormone regulation, the hypothalamus is extensively connected to limbic structures and may play functional roles in expression of emotions, sexual behavior, and memory. One prior study reported microstructural changes with OCP use within the hypothalamus using diffusion-weighted imaging [15]. The effects of OCPs on the volume of the human hypothalamus have not been reported. This gap in knowledge may be due at least in part to the lack of reliable automated methods for segmentation and volumetry of the hypothalamus [8, 9, 16–19]. Knowledge of OCP effects on the pituitary gland is similarly very limited, with only one published volumetric study [20]. In the present study, we aim to characterize alterations in hypothalamic and pituitary gland volume associated with OCP use in healthy women, using manual and/or semi-automated segmentation.

## Methods

### Participants

50 women from the New York metropolitan area were included in this analysis, enrolled prospectively between 2016 and 2018 in a supplement to a larger study of healthy athletes. A sample size calculation could not be performed a priori for this preliminary study because published data relevant to sample size determination are scarce. Participants were recruited using print and internet advertisements targeting healthy women athletes. Inclusion criteria included female sex, age 18–55, and English language fluency. Exclusion criteria included pregnancy; medical, neurological, psychiatric, or endocrine disorders; medication use including steroids; participation in organized collision sports; and use of hormonal contraception other than OCPs. Medical history was captured from a web-based questionnaire, which has been provided as supporting information (S1 Questionnaire). The study was approved by the Albert Einstein College of Medicine Institutional Review Board and complied with the Health Insurance Portability and Accountability Act. This study complied with guidelines for human studies and was conducted ethically in accordance with the World Medical Association Declaration of Helsinki. Each participant gave written informed consent.

**Fig 1:**
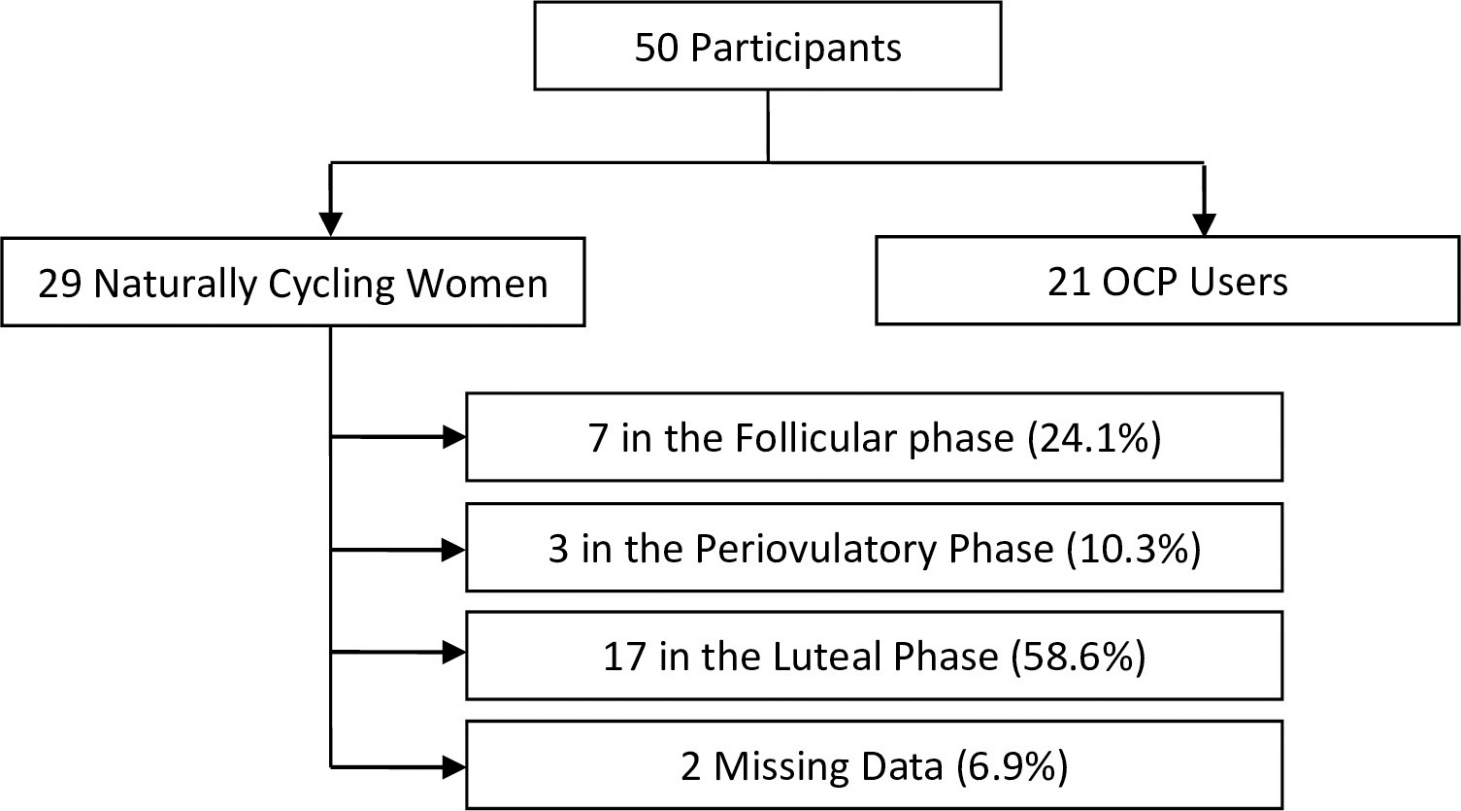
Cohort categorization based on self-reported last menstrual period.

We collected information regarding use of OCPs (yes or no), body mass index (BMI), history of smoking (yes or no) and alcohol use (yes or no) from each participant. We ascertained menstrual cycle phase, as previously described by others [21], based on participant-reported onset of the last menstrual period (LMP). Participants were classified into follicular (0–11 days from LMP), periovulatory (12–15 days from LMP), and luteal (16–36 days from LMP) phases (Fig 1). Each participant underwent brain MRI. All research took place within the Gruss Magnetic Resonance Research Center at Albert Einstein College of Medicine in Bronx, NY, USA.

### Image acquisition

MRI was performed on a 3T scanner (Achieva, Philips Healthcare, Best, The Netherlands). A 32-channel head coil (Philips Healthcare, Best, The Netherlands) was used to obtain noncontrast 3D T1-weighted MP-RAGE images in the axial orientation with the following acquisition parameters: 220 slices, 1mm^3^ voxel size, TE of 4.6 ms, TR of 9.8 ms, and flip angle of 8°.

### Image analysis

**Fig 2:**
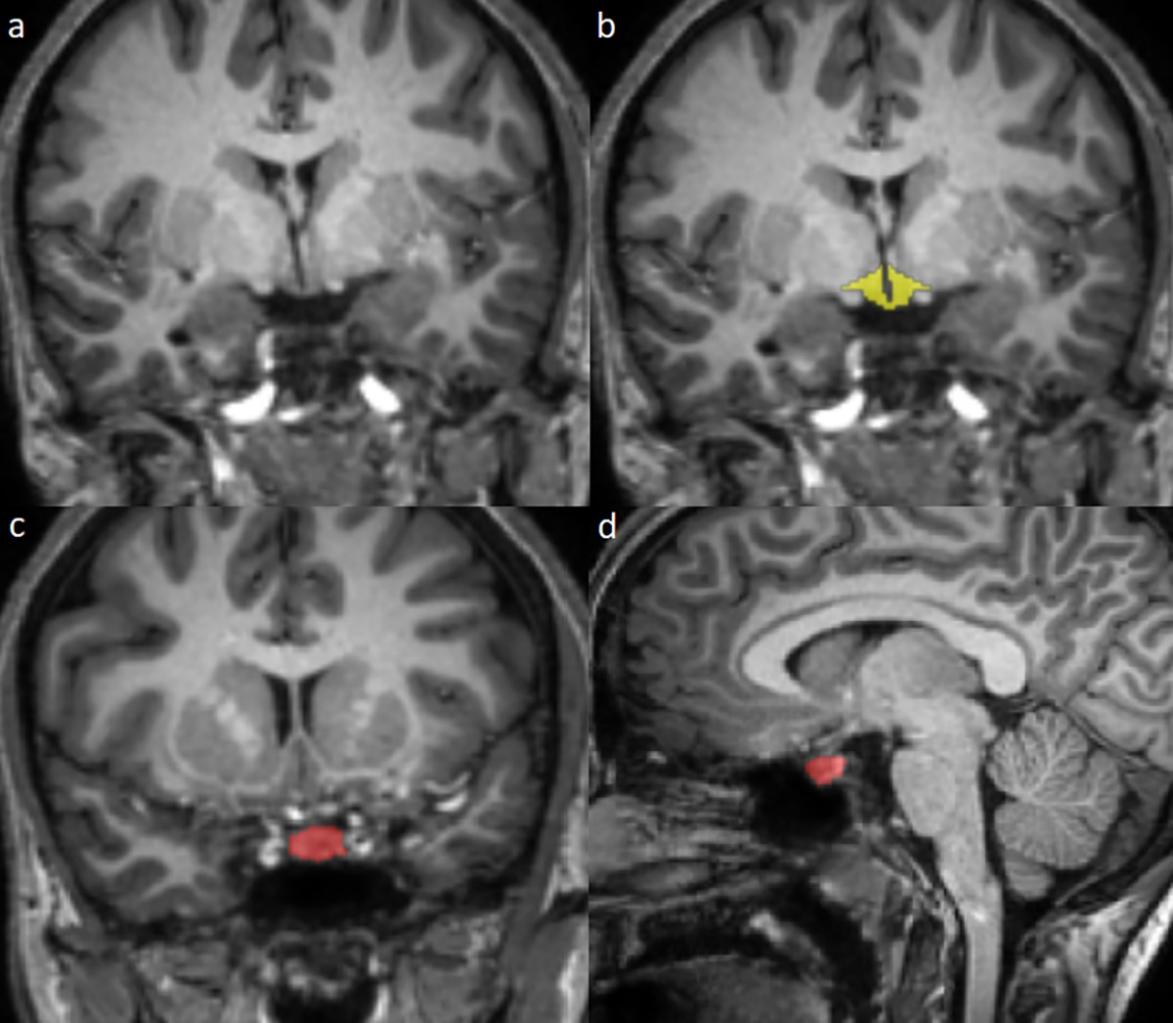
Noncontrast T1-weighted MR images of the hypothalamus and pituitary gland. The hypothalamus is shown without (**a**) and with superimposed segmentation in yellow (**b**). Segmentation of the pituitary gland (red) is shown in the coronal (**c**) and sagittal (**d**) planes.

Segmentation of the hypothalamus was based on landmarks previously determined from coronal postmortem sections [22, 23]. The anterior extent of the hypothalamus was defined as the most anterior slice on which the anterior commissure was visible. The superior border at this coronal location was defined by a straight line connecting the inferior margin of the anterior commissure to brain superior to the optic tract at its most lateral point. On subsequent slices, the superior border was defined as a line from the hypothalamic sulcus to brain superior to the optic tract at its most lateral point [16]. The posterior extent of the hypothalamus was defined as the slice that included the anterior margin of the mammillary bodies. The medial border was defined by the third ventricle. The inferior border was defined by the optic chiasm anteriorly and the interface with cerebrospinal fluid posteriorly (Fig 2A and 2B).

Segmentation of the pituitary gland was defined based on the following landmarks: sphenoid sinus anteriorly, diaphragma sellae superiorly, cavernous sinuses laterally, and dorsum sellae posteriorly. The infundibulum was not included (Fig 2C and 2D).

Images in DICOM format were loaded into ITK-SNAP version 3.6 (http://www.itksnap.org) [24], reoriented to Talairach space using a 6 parameter rigid-body transformation, and saved in NIfTI format for subsequent segmentation. Imaging review and segmentations were completed under the supervision of an American Board of Radiology Certificate of Added Qualification-certified neuroradiologist. No significant structural pathology was seen in the sellar or hypothalamic regions. Manual segmentation of the hypothalamus was performed on coronal images using the ITK-SNAP polygon tool. For the pituitary gland, a semi-automated segmentation was performed using an initial intensity threshold followed by manual edits on sagittal images using the paintbrush tool. For both hypothalamic and pituitary segmentation, orthogonal planes were referenced as needed. Five raters, blind to OCP status, performed segmentations. The cases were randomized across raters. All raters were trained on relevant anatomy, landmarks to be used for the segmentations and software tools, including review of sample segmentations, prior to beginning any work on the study data. Training and validation were undertaken on a subset of randomly selected cases, 20 for the hypothalamus and 10 for the pituitary gland. Once reliability was established, raters performed subsequent segmentations independently for the remaining cases. Total intracranial volume (tICV) was extracted automatically using FreeSurfer version 5.3 (https://surfer.nmr.mgh.harvard.edu).

### Data storage and analysis

All data reported in this paper is publicly available through the Federal Interagency Traumatic Brain Injury Repository (FITBIR) accessible at: (https://fitbir.nih.gov/). The study page on FITBIR is (https://fitbir.nih.gov/study_profile/220). Statistical analysis was performed using SPSS 25 (IBM Corp.; Armonk, NY). The inter-rater intraclass correlation coefficient (ICC) was calculated to assess inter-rater agreement using a two-way random model with measures of absolute agreement. Student’s T-test and Pearson’s chi-square test were used to compare continuous and categorical demographic variables, respectively. General linear models were used to test for differences in hypothalamic and pituitary gland volume between OCP and NCW groups. Assumptions for linear regression were assessed and met including normality, multicollinearity, independence, linearity, homoscedasticity, and outliers. The covariates used in these models included OCP use (yes/no), tICV, BMI, smoking history (yes/no), and alcohol use (yes/no). Significance is reported at p<0.05. Exploratory analyses were performed to assess the effect of menstrual cycle phase on hypothalamic and pituitary volume as well as to compare volumes among OCP users with NCW imaged at different phases of the menstrual cycle. One-way analysis of covariance (ANCOVA) was performed while controlling for tICV with four groups: three menstrual phase subgroups of NCW (follicular, periovulatory, and luteal phases) and OCP users. Post-hoc analysis was performed with results adjusted to account for multiple testing using the Bonferroni method.

## Results

### Participant characteristics

50 women (mean age ± standard deviation; 22.1 ± 3.7 years) comprising of 21 OCP users (age range, 19–29) and 29 NCW (age range, 18–36) were enrolled. There was no significant difference in age, BMI, and smoking history between NCW and OCP users (Table 1). More OCP users consumed alcohol when compared to NCW (p = 0.001), but no participant consumed more than 7 drinks per week. For the NCW, 7 (24.1%) participants were classified as follicular phase, 3 (10.3%) were classified as periovulatory phase, and 17 (58.6%) were classified as luteal phase. For 2 participants (6.9%), information regarding the LMP was not available.

**Table 1:** Demographic features of the cohort.

	OCP Users (n = 21)	Naturally Cycling Women (n = 29)	p-value
Age (years; mean ± SD)	23.0 ± 3.1	21.4 ± 4.0	0.12^a^
BMI (kg/m^2^; mean ± SD)	22.3 ± 3.2	23.0 ± 3.7	0.52^a^
History of smoking	1/21 (4.8%)	2/29 (6.9%)	0.75^b^
History of alcohol use	19/21 (90.5%)	13/29 (44.8%)	**0.001**^b^

a: Student’s T-test

b: Pearson’s Chi-squared test

### Segmentation reliability

We found good agreement of the segmentation results among all five raters in our study. The ICC was 0.86 for hypothalamic segmentation and 0.78 for pituitary segmentation.

### OCP and hypothalamic and pituitary gland volumes

The mean volume for the hypothalamus was 683.4 ± 93.0 μL for OCP users and 730.4 ± 77.7 μL for NCW (difference of 47 μL). The mean volume for the pituitary gland was 704.4 ± 127.2 μL for OCP users and 803.1 ± 106.4 μL for NCW (difference of 98.7 μL).

We found that OCP use was associated with both smaller total hypothalamic (B = -81.2 ± 24.9, p = 0.002) and pituitary gland volume (B = -81.2 ± 38.7, p = 0.04) when compared to NCW. The results of our regression models are shown in Tables 2 and 3.

**Table 2:** Regression model for hypothalamic volume.

Covariate	B ± SE	β	p-value
Birth Control	-81.2 ± 24.9	-0.5	**0.002**
TIV	2.3 x 10^−4^ ± 7.6 x 10^−5^	0.4	**0.004**
Age	-1.6 ± 3.2	-0.1	0.62
BMI	0.1 ± 3.3	0.005	0.97
Alcohol	45.2 ± 26.7	0.3	0.10
Smoking	-57.9 ± 45.8	-0.2	0.21

**Table 3:** Regression model for pituitary volume.

Covariate	B ± SE	β	p-value
Birth Control	-81.2 ± 38.7	-0.3	**0.04**
TIV	5.8 x 10^−5^ ± 1.2 x 10^−4^	0.1	0.62
Age	-2.3 ± 5.0	-0.1	0.66
BMI	9.1 ± 5.1	0.3	0.08
Alcohol	-26.9 ± 41.6	-0.1	0.52
Smoking	-27.4 ± 71.2	-0.1	0.70

### Exploratory subgroup analyses: Menstrual cycle phase effects

On testing for differential effects of menstrual cycle phase, we found significant divergence of hypothalamic volume among the groups (F(4,44) = 3.02, p = 0.03). Post-hoc testing confirmed smaller hypothalamic volume in OCP users when compared to NCW in the luteal phase (p = 0.01), but not to the other phases. There was no significant difference in total hypothalamic volume between the different phases of the menstrual cycle in NCW.

We did not find a significant divergence of pituitary volume among the groups (F(4,44) = 2.5, p = 0.06). There was no significant volume difference between OCP users and NCW in each different phase of the menstrual cycle. We found no significant effect of menstrual cycle phase on pituitary gland volume among NCW at different menstrual cycle phases.

## Discussion

Our results indicate that OCP use is associated with smaller hypothalamic and pituitary volumes in healthy women. These findings add to available knowledge on the structural effects of OCPs on the brain. Most notably, effects of OCP use on direct measurements of hypothalamic volume have not been reported. Pertinent data on the pituitary gland is extremely limited. In the only study published to date, pituitary volume in OCP users was lower by 60 μL compared to NCW, which is congruent with our findings. However, the participants in that study underwent MRI examinations for evaluation of neurological symptoms and diseases [20]. Given the young age of our cohort, absence of significant medication use or medical conditions including neurological, psychiatric, and endocrine disorders, our findings are less likely to be influenced by confounding factors that may affect brain volumes and more likely to represent effects that are solely due to OCP use. Moreover, neither smoking history nor alcohol use, when included in our linear regression model, reached statistical significance as covariates impacting hypothalamic and pituitary volumes.

Although our primary aim was not to characterize the role of menstrual cycle phase on brain structure in relationship to OCP use, we performed secondary exploratory subgroup analyses, which suggest a mechanistic explanation for our findings. Specifically, hypothalamic volume in OCP users was smaller when compared to the subgroup of NCW in the luteal phase, but not compared to those in the other menstrual phases. OCPs suppress endogenous production of ovarian sex hormones [25–27] and serum concentrations of estradiol and progesterone are thus substantially higher during the luteal phase in NCW compared to the active pill phase in those taking OCPs. On the other hand, hormone levels do not differ between NCW in the follicular phase and OCP users during the inactive pill phase [11]. In our study, a large proportion of NCW were in the luteal phase at the time of imaging. Thus, from a biochemical perspective, smaller hypothalamic volume in OCP users may be driven by this pronounced difference in hormone levels conferred by the active OCP. We did not detect a similar relationship for the pituitary gland. This discrepancy might implicate the hypothalamus as the primary site of central OCP effect, but may also stem from reduced sensitivity of these exploratory analyses due to small subgroup sample sizes. The results of these subgroup analyses of volumetric effects across the menstrual cycle must therefore be considered exploratory and motivate future investigation.

Estrogen and progesterone have been shown to exhibit trophic effects in various regions of the brain by modulating dendritic spine number and synapse density [28–30]. In this context, the low hormone concentrations seen in OCP users, particularly during the active pill phase, may manifest as tissue volume reduction, which would be concordant with our findings. However, the effects of estrogen and progesterone on the brain are complex, entailing more than simple up- and down-regulation [31]. Rodent studies have revealed that chronic treatment with progesterone reverses its short-term trophic effects on the hippocampus and may downregulate estrogen-induced synapses when co-administered with estrogen [32]. The relevance of these preclinical findings must also be considered in light of incomplete knowledge on the neuronal targets of exogenous sex steroids in humans, which are largely unknown [3].

The structural effects of OCPs on the hypothalamic-pituitary system we found in this study cannot be directly taken as evidence of any specific cellular mechanism. The volumetric effects, however, could be consistent with inhibition of folliculogenesis by OCPs and thus may be related to potential downstream neurobehavioral effects from OCP use. OCPs prevent ovulation via negative feedback on the hypothalamic-pituitary-ovarian axis, blocking secretion of GnRH from the hypothalamus and FSH and LH from the pituitary [27, 33]. Our volumetric findings in OCP users may provide a structural correlate to this phenomenon. A prior MRI study demonstrated a higher apparent diffusion coefficient (ADC) within the hypothalamus during the inactive pill phase compared to the active pill phase in OCP users [15]. The ADC is a measure of the magnitude of water diffusion within tissue, computed from diffusion-weighted MRI. The higher ADC is consistent with microstructural changes that facilitate diffusion, such as decrease in cell density. The lower ADC during the active pill phase, consistent with higher tissue density, on the other hand, may occur due to synaptogenesis or change in neural morphology. It is difficult, however, to directly compare the findings of this within-subjects analysis based on a measure of microstructural features (ADC) to our results, which compare macrostructural hypothalamic volume of OCP users to that of NCW. The hypothalamus not only regulates endocrine function, but is also central to sleep regulation and extensively connected to limbic and prefrontal systems that subserve emotion regulation, memory, and other aspects of cognition. Although this study does not directly address the question of functional effects, it is plausible that change in hypothalamic may be related changes in neurobehavioral function. Potential for associated functional effects is an area of interest for future studies since, at the current time, the literature on functional and emotional effects of OCPs is mixed [3, 34–37].

In our exploratory analyses, we found no difference in hypothalamic and pituitary volumes for NCW based on menstrual phase. Our finding regarding the pituitary is concordant with that of a prior study [20]. Larger right hypothalamic volume has been demonstrated during menstruation, compared to the periovulatory phase, in women with primary dysmenorrhea [38]. However, no direct volumetric studies on the hypothalamus in healthy women are available for comparison. It remains unknown whether structural changes of the hypothalamus are relevant to disease states only or also play a role in normal functioning.

Our study was performed using manual segmentation, which is considered the gold-standard approach for brain volumetry. However, our findings should be interpreted in light of several limitations. Although this is the largest study of hypothalamic and pituitary volumetry in healthy women athletes, sample size still limits sensitivity and generalizability. The cross-sectional design of this study precludes confirmation of causal connections between OCP use and hypothalamic or pituitary volume. We tested the class-level effect of current OCP use, but were not able to address potentially relevant factors such as duration of current or prior OCP use, androgenicity of OCPs, parity status, and pill phase [3, 4, 11]. We cannot exclude bias stemming from self-selection as well as differences in age, cycle phase, and pill phase that could have influenced findings. We cannot entirely rule out dependence of the brain volume changes on participant hydration status, although we have not found published data that support a hypothalamic volume change due to hydration that reaches the magnitude of the effects we report related to OCP. Moreover, an exceptionally high degree of systematic confounding across NCW and OCP users would be required to explain our findings. We believe this is unlikely. We categorized menstrual cycle phase based on self-reported LMP, which has been widely used in the literature, but may be subject to recall bias. Reliability across raters for our segmentation results were very good, but not in the excellent range. We note that our approach using multiple raters augments the potential for variance across raters, but similarly protects against the likelihood of bias due to chance agreement in the setting of fewer raters. It is therefore more likely that we underestimate the effects that we report and are less susceptible to false positive inference. We did not include rater heterogeneity as a parameter in our models, which may add noise to the parameter estimates. In regard to the pituitary gland, image artifacts common to the sella region may subject the pituitary segmentations to error as suggested by the lower reliability of pituitary segmentation across raters compared to the hypothalamus.

## Conclusions

OCP use in healthy women is associated with smaller hypothalamic and pituitary volumes, which may be related to disruption of known trophic effects of ovarian sex hormones, although our study design precludes confirmation of any specific mechanism. Future studies to investigate hypothalamic volume with respect to serum ovarian sex hormone levels at multiple points during the menstrual and OCP pill phase cycles, in conjunction with neurobehavioral assessments, are warranted to further delineate differential effects of endogenous and exogenous sex steroids on brain structure and function.

## Supporting information

S1 Questionnaire Medical and psychiatric history questionnaire. (DOCX)
