# Supplementary material for: Oral contraceptive use is associated with smaller hypothalamic and pituitary gland volumes in healthy women: A structural MRI study
Source: PLoS One. 2021 Apr 21;16(4):e0249482. doi: 10.1371/journal.pone.0249482 (PMC8059834; doi:10.1371/journal.pone.0249482)
Supplement: S1 Questionnaire — (DOCX) [file pone.0249482.s001.docx]

No Yes - I was never treated

**If you have ever been diagnosed with the following psychiatric conditions, please indicate:**

Yes - I have been treated in the past

Yes - I am currently being treated

| Bipolar Disorder | 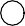 | 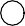 | 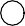 | 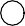 |
| --- | --- | --- | --- | --- |
| Depression | 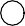 | 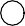 | 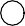 | 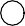 |
| Anxiety | 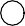 | 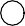 | 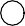 | 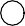 |
| Psychosis | 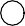 | 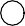 | 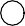 | 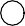 |
| Schizophrenia | 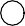 | 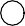 | 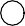 | 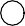 |

Have your parents, siblings, or children been Yes


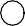

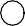


diagnosed with any medical, neurological, or No psychiatric problems?

If so, please specify which medical, neurological, or psychiatric problems:

Have you ever been told that you have a neurological No


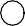

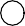

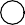

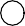


condition? Yes - I was never treated

Yes - I have been treated in the past Yes - I am currently being treated

If you have been told that you have a neurological condition, please specify the condition:

Do you have any other (non-neurological) medical No


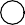

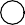

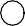

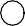


problems? Yes - I was never treated

Yes - I have been treated in the past Yes - I am currently being treated

Please specify your (non-neurological) medical problem(s):

Do you have problems with any of the following (please check off all of those which apply):

Hands/use of hands

Vision (e.g. color perception, visual acuity that is not corrected by glasses or contact lenses) Hearing

None of the above

Do you have/have you ever been told that you have a history of any of the following (please check all of those which apply):

Heart disease Diabetes

High blood pressure Stroke

None of the above

Are you currently taking any medications? Yes No


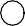

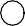


Please list all medications you are currently taking:

Have you ever been hospitalized? Yes No


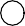

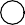


If so, please specify what you were hospitalized for:
